# Comparative Analysis of Carotenoid Profiles and Biosynthetic Gene Expressions among Ten Plum Cultivars

**DOI:** 10.3390/plants12142711

**Published:** 2023-07-21

**Authors:** Honghong Deng, Xingyu Long, Xi Wang, Yang Wang, Changqing Pang, Hui Xia, Dong Liang, Huifen Zhang, Xian Luo, Jin Wang, Xiulan Lv, Qunxian Deng

**Affiliations:** College of Horticulture, Sichuan Agricultural University, Chengdu 611130, China; denghh@sicau.edu.cn (H.D.); 20173309@stu.sicau.edu.cn (X.L.); 2018305019@stu.sicau.edu.cn (X.W.); 2020105004@stu.sicau.edu.cn (Y.W.); 2022305090@stu.sicau.edu.cn (C.P.); xiahui@sicau.edu.cn (H.X.); liangeast@sicau.edu.cn (D.L.); 14339@sicau.edu.cn (H.Z.); 13996@sicau.edu.cn (X.L.); 14224@sicau.edu.cn (J.W.); 10998@sicau.edu.cn (X.L.)

**Keywords:** biosynthetic gene expression, carotenoids, content and compositions, HPLC, plums

## Abstract

Plums are good sources of various bioactive phytochemical compounds such as vitamins, anthocyanins, and carotenoids, whereby all of which are noted for multiple potential health benefits. However, knowledge regarding plum carotenoid profiles remains limited. Hence, the total and individual carotenoids in the edible parts (skin and flesh) of ten plum cultivars were determined using a spectrophotometer and high-performance liquid chromatography–diode array detection, respectively. Total and individual carotenoid contents in skin were significantly higher (*p* < 0.05) than those in flesh among all plum cultivars tested. The cultivars with the highest content of total carotenoids in skin were Naili (36.73 μg/g FW), followed by Yinhongli (21.81 μg/g FW) and Yuhuangli (19.70 μg/g FW), with the lowest in Angeleno (8.97 μg/g FW). Lutein, zeaxanthine, β-cryptoxanthin, α-carotene, and β-carotene were the major types of carotenoids detected, with lutein and β-carotene being the predominant constituents of the skin and flesh tissues, respectively. Lutein, zeaxanthine, and total carotenoid contents were positively correlated with the expressions of *PSY*, *LCYB*, and *LCYE*, and negatively correlated with the expressions of *PDS* and *CRTISO*. Characterizing the carotenoid profiles and investigating variations in carotenoid biosynthetic gene expressions among plum cultivars are crucial for advancing genetic improvements in plums.

## 1. Introduction

Plums (*Prunus* spp.), belonging to the subfamily Prunoideae and family Rosaceae, are a large and diverse group of stone fruit species widely consumed in both fresh and processed forms worldwide. This is particularly true in Europe, Asia, and the Americas [1,2,3]. Among the various types, the European plum (*P. domestica* L., 2n = 6x = 48) and the Chinese plum (also known as Japanese plum, *P. salicina* Lindl., 2n = 2x = 16) hold significant commercial importance, with numerous varieties within each category [4,5]. In recent times, plums have attracted increasing attention due to their balanced taste and bioactive-dense compounds, such as dietary fibers, micronutrients, vitamins, phenols, and carotenoids. These kinds of compounds are noted for having multiple potential health benefits [6,7,8].

Among them, carotenoids are natural isoprenoid pigments that produce distinctive colors (yellow, orange, and some reddish colors) in plant leaves, fruits, flowers, and vegetables for attracting pollinators, seed dissemination, and increased consumer appeal [9]. These properties make carotenoids an important indicator of food quality, as well as an economic target for the food industry and related industries [9,10]. Apart from making the fruit and plant attractive, carotenoids can serve important roles in various biological processes of plants, such as photosynthesis, photoprotection, phytohormone synthesis, and signaling [10]. Moreover, carotenoids could provide important precursors for vitamin A and phytohormones, such as abscisic acids and strigolactones. Carotenoids have been widely recognized as powerful antioxidants because of the potential to treat vitamin A deficiency, alleviate various chronic diseases (cancer and cardiovascular diseases), and promote immune functions [11]. It is worth nothing that these natural antioxidant compounds cannot be synthesized by humans and other primates themselves; hence, they obtain carotenoids via dietary supplementation with plant products [12]. Horticulture plants, particularly fresh fruits and vegetables, constitute the most important dietary sources of carotenoids for human beings [13].

**Figure 1:**
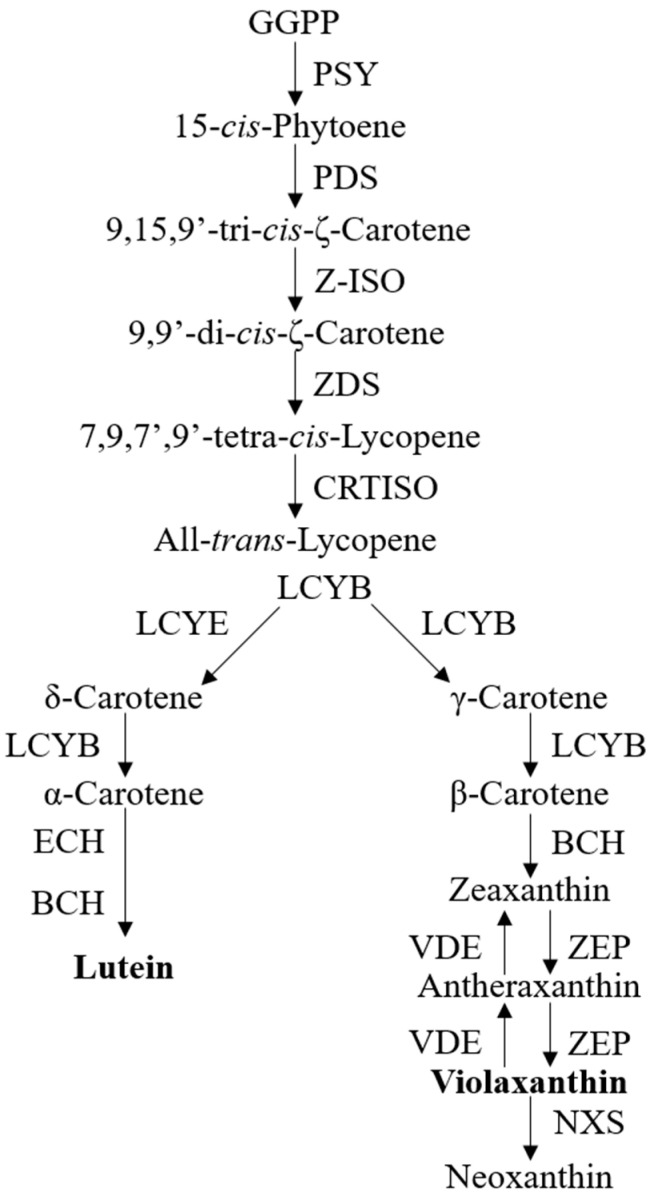
The current known pathway for carotenoid biosynthesis. Abbreviations: geranylgeranyl diphosphate, GGPP; phytoene synthase, PSY; phytoenede saturase, PDS; ζ-carotene isomerase, Z-ISO; ζ-carotene desaturase, ZDS; carotene isomerase, CRTISO; lycopene ε-cyclase, LCYE; lycopene β-cyclase, LCYB; β-carotene hydroxylase, BCH; ε-hydroxylase, ECH; zeaxanthin epoxidase, ZEP; violaxanthin de-epoxidase, VDE.

Due to the associations of carotenoids with plant pigments, biological processes, and human health benefits, the understanding of carotenoid metabolism and regulation has advanced rapidly [9,10,14]. The carotenoid biosynthetic pathway begins with the head-to-head condensation of two geranylgeranyl diphosphate (GGPP) molecules to form the first carotenoid product, 15-*cis*- phytoene, by phytoene synthase (PSY) [9], which is thus considered as a crucial rate-limiting enzyme of carotenoid biosynthesis in many plant species [15]. The colorless phytoene is desaturated and isomerized sequentially with phytoenede saturase (PDS) [15], ζ-carotene isomerase (Z-ISO) [16], ζ-carotene desaturase (ZDS) [17], and carotene isomerase (CRTISO) [18] to produce a red-colored all-*trans*-lycopene. At this point, the cyclization of lycopene is then divided into an α- and β-branch, catalyzed by lycopene ε-cyclase (LCYE) and/or lycopene β-cyclase (LCYB), to form orange α-carotene and β-carotene, respectively. The subsequent hydroxylation of α-carotene and β-carotene by β-carotene hydroxylase (BCH) and ε-hydroxylase (ECH) yields yellow xanthophyll carotenoids, i.e., lutein in α-branch and zeaxanthin in β-branch. Zeaxanthin can be epoxidized by zeaxanthin epoxidase (ZEP) to violaxanthin and this reaction can be reversed back by violaxanthin de-epoxidase (VDE) [9,10,14]. The conversion of violaxanthin into neoxanthin, which is the final carotenoid product, concludes the core carotenoid biosynthetic pathway [10]. Figure 1 provides a concise visual representation of the carotenoid biosynthesis pathway in most species (Figure 1).

Previous research has mostly focused on the phenolic compounds of plums, particularly anthocyanins [7,19,20]. However, there is a noticeable lack of literature on the characterization of carotenoids. Consequently, the objective of this work is to evaluate the composition and content of the carotenoids present in the edible parts (i.e., skin and flesh) of ten distinct plum cultivars, which exhibit varying fruit color and flavor profiles, all grown within the same geographical location. This investigation specifically focuses on determining the total carotenoids and identifying the individual carotenoid compounds. Furthermore, the expression of carotenoid biosynthetic genes was also explored. The correlation between the carotenoid content and the expression of related genes was revealed. The results of this study will help us to select plum cultivars with the highest amount of total and specific individual carotenoids. A better understanding of the variability in the carotenoid content of plums provides a crucial foundation for future genetic breeding targeting plum cultivars with higher carotenoid levels.

## 2. Results

### 2.1. Comparative Analyses of Fruit Weight, Shape Index, and Color of Ten Plum Cultivars

The various fruit phenotypes of the ten plum cultivars are presented in Table 1. Based on the visual estimation, the tested cultivars exhibit the distinct color: Yuhuangli displays both dark yellow skin (epicarp, the outermost layer) and flesh (mesocarp, the middle layer) when ripe. Black Amber, on the other hand, possesses purple or black skin and yellow flesh. Naili exhibits firm and green skin and flesh. Black Diamond Ooishi-wase and Angeleno share similarities in terms of their skin, which is a combination of purple and black, and their yellow flesh (Table 1).

**Figure 2:**
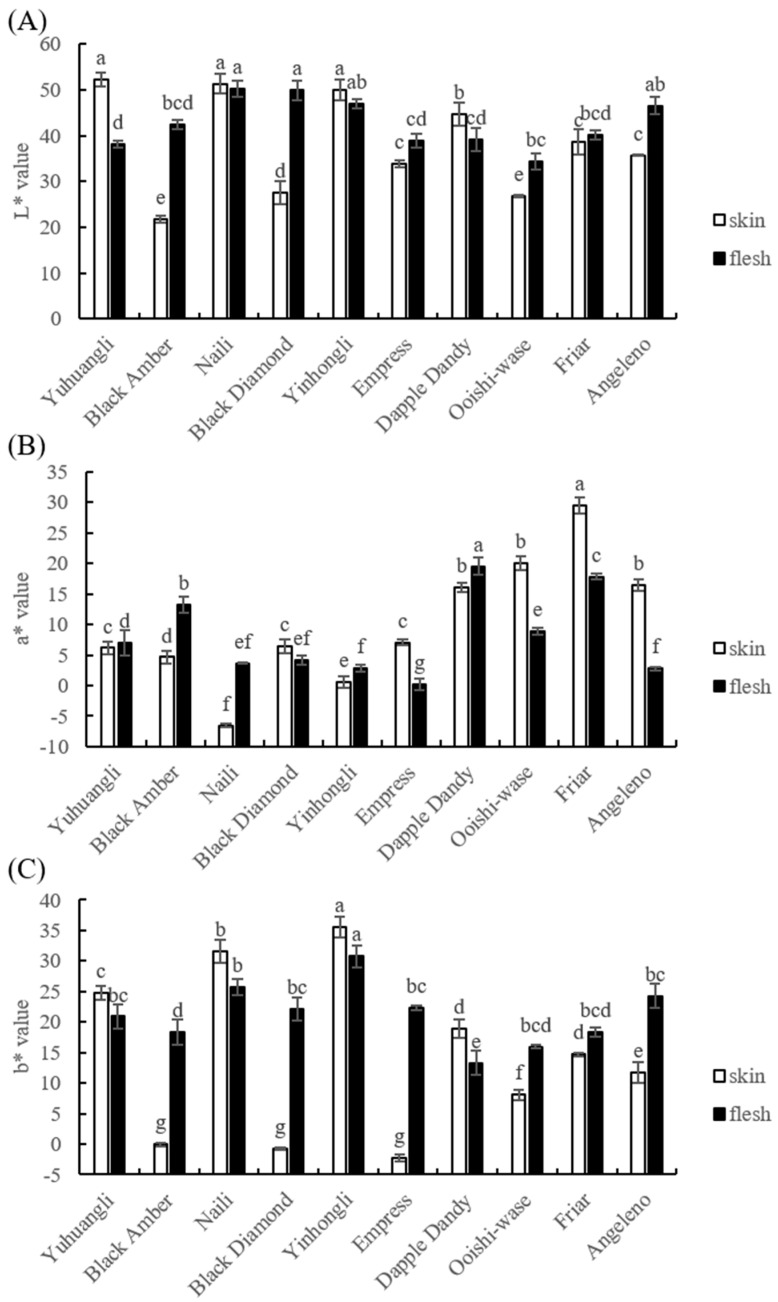
Comparative analysis of the color index of the skin (epicarp, the outermost layer) and flesh (mesocarp, the middle layer) of ten plum cultivars. (**A**) L* value, (**B**) a* value, and (**C**) b* value. The different letters on the bars indicate significant difference (*p* < 0.05).

Precise color measurements using a colormeter are depicted in Figure 2 and the L*, a*, and b* values of the skin and flesh were determined. Yuhuangli, Naili, and Yinhongli contained significantly higher (*p* < 0.05) levels of L* values in the skin, indicating their bright colors, in contrast to Black Amber and Ooishi-wase which displayed significantly lower (*p* < 0.05) L* values in the skin, indicating their dull colors. Furthermore, Naili and Black Diamond demonstrated significantly higher (*p* < 0.05) L* values in the flesh compared to other cultivars. In addition, Yinhongli and Angeleno exhibited slightly higher L* values in the flesh compared to Black Amber and Friar, while Yuhuangli displayed significantly lower (*p* < 0.05) L* values compared to other cultivars.

In terms of skin color, the cultivar Friar had the highest a* value, with Dapple Dandy, Ooishi-wase, and Angeleno following suit, while Naili had a negative a* value. Regarding flesh color, Dapple Dandy had the highest a* value, with Black Amber, Friar, and Yuhuangli trailing behind, whereas Angeleno had the lowest a* value.

Yinhongli had the highest b* value in skin, followed by Naili, Yuhuangli, Dapple Dandy, Friar, and Angeleno, whereas Black Amber, Black Diamond, and Empress had a negative b* value. In terms of flesh color, Yinhongli had the highest b* value, followed by Naili, whereas Dapple Dandy had the lowest b* value.

**Figure 3:**
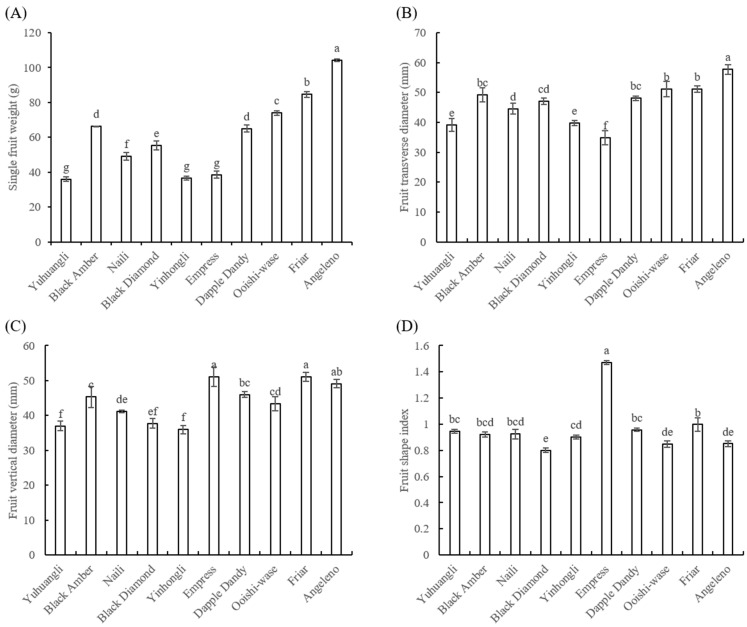
Comparative analysis of the single fruit weight (**A**) and fruit shape index (**B**–**D**) of ten plum cultivars. The different letters on the bars indicate significant difference (*p* < 0.05).

The cultivar Angeleno (104.26 g) exhibited the highest single fruit weight, followed by Friar (84.59 g), and the lowest weight was observed in Yuhuangli (36.04 g) (Figure 3A). Angeleno (57.75 mm) also displayed the highest transverse diameter, while the Empress (34.79 mm) had the lowest value (Figure 3B). The cultivars Friar (51.07 mm) and Empress (51.1 mm) exhibited significantly higher (*p* < 0.05) fruit vertical diameters compared to the other cultivars (Figure 3C). The fruit shape index ranged from 0.85 to 0.95, except for Express (1.47) and Black Diamond (0.80) (Figure 3D).

### 2.2. Comparative Analyses of Total and Individual Carotenoids of Ten Plum Cultivars

**Figure 4:**
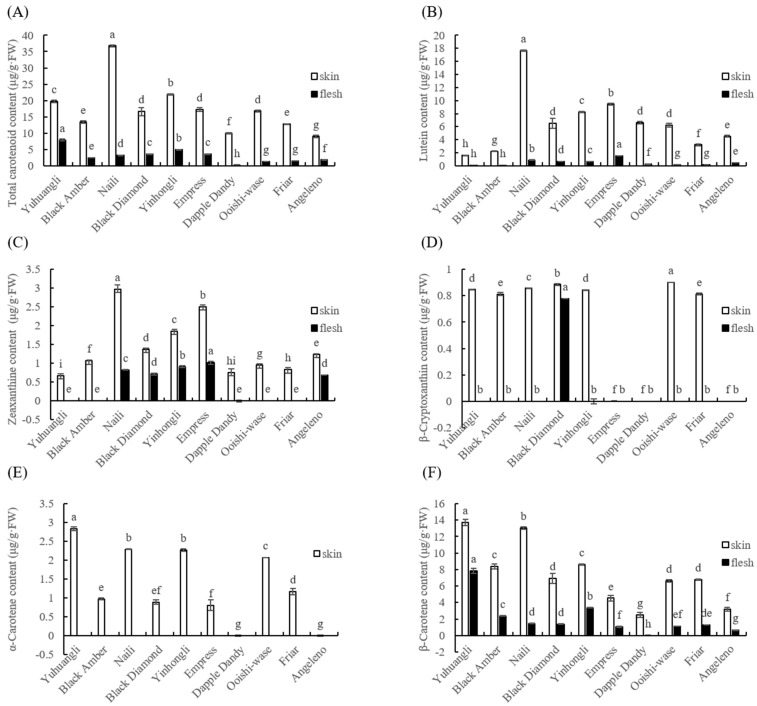
Comparative analysis of the total (**A**) and individual (**B**–**F**) carotenoids of ten plum cultivars. The different letters on the bars indicate significant difference (*p* < 0.05).

Both the outermost skin and the middle flesh of the plum fruit are edible; thus, the total and individual carotenoid content in the skin and flesh of ten plum cultivars were comparatively determined. Among all of the plum cultivars tested, the total and individual carotenoid contents in skin tissues were found to be significantly higher (*p* < 0.05) than those in flesh samples (Figure 4). This suggested that there is a notable difference in the ability of plums to accumulate carotenoids upon maturity depending on cultivars. Specifically, the cultivars Naili (36.73 μg/g FW), Yinhongli (21.81 μg/g FW), and Yuhuangli (19.70 μg/g FW) exhibited the highest total carotenoid content in their skin, while Angeleno (8.97 μg/g FW) had the lowest. On the other hand, Yuhuangli (7.92 μg/g FW) was the cultivar with the highest carotenoid content in the flesh, followed by Yinhongli (4.95 μg/g FW), with Dapple Dandy (0.25 μg/g FW) having the lowest (Figure 4A).

The content and composition of individual carotenoids were analyzed using HPLC. The carotenoids identified in the ten plum cultivars included lutein, zeaxanthine, β-cryptoxanthin, α-carotene, and β-carotene. Interestingly, the composition of carotenoids varied significantly between the skin and flesh tissues, even within the same cultivar. Lutein was found to be the most abundant carotenoid in the most cultivars, with an average content of 6.63 μg/g FW in the skin (ranging from 1.61 μg/g FW in Yuhuangli to 17.62 μg/g FW in Naili) and 0.50 μg/g FW in the flesh (ranging from 0.08 μg/g FW in Yuhuangli and Black Amber to 1.51 μg/g FW in Empress) (Figure 4B). The skin of Naili (2.97 μg/g FW) exhibited the highest concentration of zeaxanthine, followed by Empress (2.51 μg/g FW) and Yinhongli (1.86 μg/g FW), while Yuhuangli (0.68 μg/g FW) had the lowest concentration. In the flesh of Yuhuangli, Black Amber, Dapple Dandy, Ooishi-wase, and Friar, the zeaxanthin concentration was below the limit of quantification. Empress (1.02 μg/g FW) had the highest zeaxanthine concentration in the skin, while Angeleno (0.69 μg/g FW) had the lowest (Figure 4C). Ooishi-wase (0.90 μg/g FW) had the highest level of β-cryptoxanthin, whereas Empress, Dapple Dandy, and Angeleno did not exhibit any detectable β-cryptoxanthin in their skin tissues.

Amongst the flesh samples, the presence of β-cryptoxanthin was exclusively observed in Black Diamond (0.78 μg/g FW) (Figure 4D). α-Carotene, on the other hand, was only detected in the skin tissues, with the highest content found in Yuhuangli (2.83 μg/g FW), followed by Naili (2.29 μg/g FW) and Yinghongli (2.26 μg/g FW), and the lowest was observed in Empress (0.80 μg/g FW). α-Carotene was not detected in the skin tissues of Dapple Dandy and Angeleno (Figure 4E). β-Carotene was the highest in both the skin (13.73 μg/g FW) and flesh (7.84 μg/g FW) tissues of Yuhuangli, followed by Naili (13.00 μg/g FW) and Yinghongli (3.34 μg/g FW), and it was the lowest in Dapple Dandy (2.50 μg/g FW) and Angeleno (0.66 μg/g FW) (Figure 4F).

### 2.3. Comparative Analyses of Gene Expressions for Carotenoid Biosynthesis of Ten Plum Cultivars

**Figure 5:**
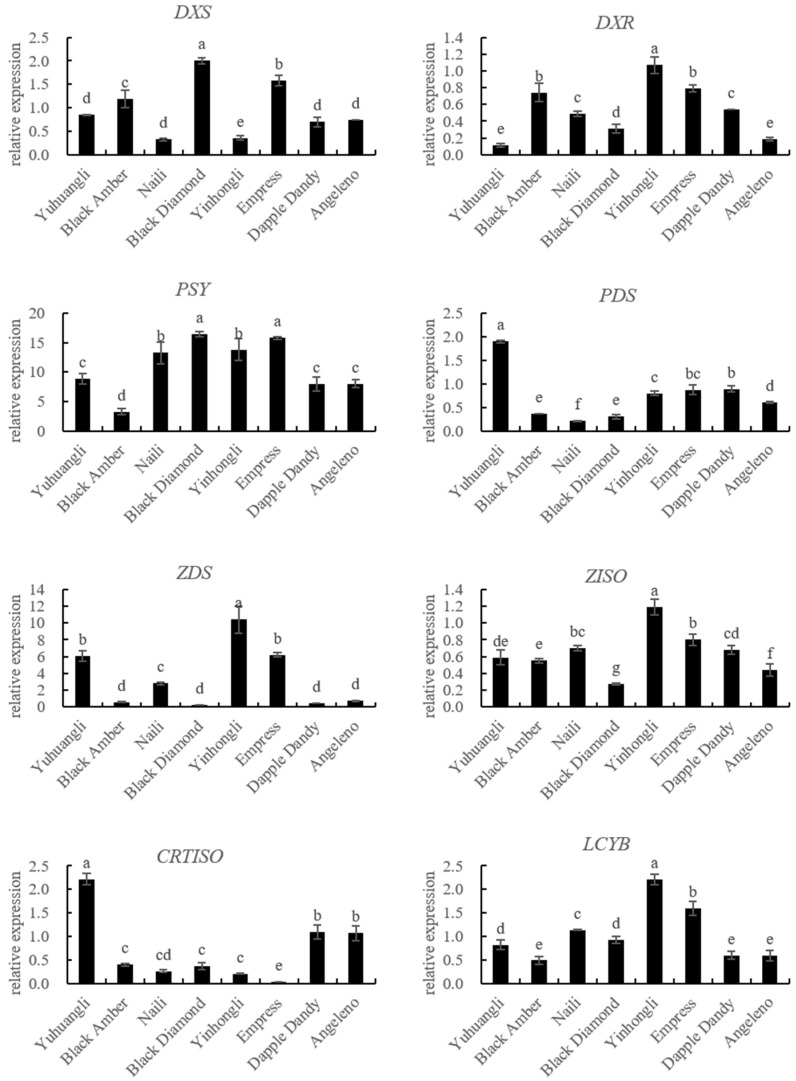

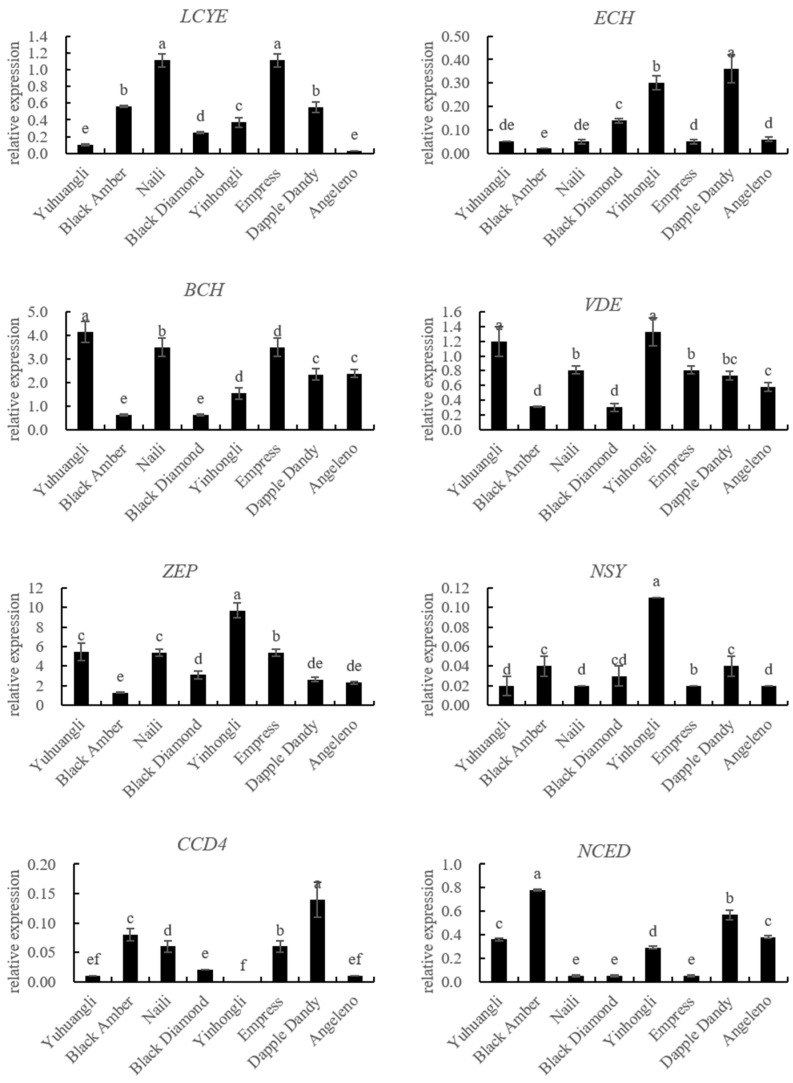
Comparative analyses of gene expressions for carotenoid biosynthesis of ten plum cultivars. The different letters on the bars indicate significant difference (*p* < 0.05).

The gene expressions related to carotenoid biosynthesis in the ten plum cultivars were simultaneously investigated using qRT-PCR (Figure 5). The correlations between carotenoids and the gene expressions related to carotenoid biosynthesis are summarized in Table 2. Specifically, the lutein content exhibited significant correlations with the expressions of *PSY* (r = 0.574 **), *LCYE* (r = 0.801 **), *PDS* (r = −0.484 *), and *CRTISO* (r = −0.566 **). The zeaxanthine content showed significant correlations with the expressions of *PSY* (r = 0.614 **), *LCYB* (r = 0.572 **), *LCYE* (r = 0.699 **), *ZEP* (r = 0.589 **), *PDS* (r = −0.464 *), and *CRTISO* (r = −0.700 **). In all of the tested cultivars, there were notable negative correlations between the β-cryptoxanthin content and the expressions of *CCD4* (r = −0.521 **) and *NCED* (r = −0.570 **). Additionally, we observed significantly positive correlations between the α-carotene content and the expressions of *ZDS* (r = 0.641 **), *VDE* (r = 0.616 **), *LCYB* (r = 0.447 *), and *BCH* (r = 0.480 *), and a negative correlation between the α-carotene content and the expression of *CCD4* (r = −0.489 *). The β-carotene content was significantly correlated with the expressions of *BCH* (r = 0.500 *), *ECH* (r = −0.420 *), *CCD4* (r = −0.437 *), and *NCED* (r = −0.429 *). Overall, the total carotenoid content demonstrated a significantly positive correlation with *PSY* (r = −0.444 *), *LCYB* (r = 0.432 *), and *LCYE* (r = 0.666 *), while displaying a significantly negative correlation with *NCED* (r = −0.407 *).

## 3. Discussion

The consumption of fresh fruits and vegetables offers numerous health-related benefits, whereby some of which can be attributed to the presence of carotenoids [13]. Plums are an excellent source of carotenoids. Despite extensive research on the phenolic compounds of plums, particularly anthocyanins [7,19,20], the investigation of carotenoid biosynthesis and its regulation in plum fruit has been neglected. In this study, the content and composition of carotenoids and the gene expressions related to carotenoid biosynthesis in the edible parts (skin and flesh) of ten plum cultivars were compared.

The species, varieties, geographical locations, and environmental factors, such as light exposure, soil composition, and climatic conditions, are the most influential factors affecting the concentration and composition of carotenoids [21,22]. Furthermore, it is noteworthy that the carotenoid profiles may differ among different parts of the same plant; for example, the carotenoid content and compositions may vary qualitatively and especially quantitatively in different parts of the same plant [21]. In the present study, all of the plum trees were subjected to identical agronomic and climatic conditions, as well as horticultural management and analytical parameters. The observed differences and similarities were solely attributed to the cultivar.

It was found that the total carotenoid content of the skin was significantly higher (*p* < 0.05) in comparison to that of the flesh in each plum cultivar (Figure 4). This result was consistent with a previous study [21]. The plum cultivars exhibited significant variation in total carotenoid content, with the green-yellow cultivar Naili displaying the highest content, followed by Yinhongli and Yuhuangli, while the purple cultivar Angeleno exhibited the lowest content (Figure 4A). These findings contradict the results reported by Diaz-Mule et al. [23], because they demonstrated that the red-pigmented plum cultivars like Larry Ann and Black Amber had higher carotenoid content in both skin and flesh tissues compared to the yellow pigmented plum cultivars like Golden Globe and Golden Japan. Vizzotto et al. [24] reported that the plum cultivars with light-colored flesh had slightly higher carotenoid content compared to the red-fleshed cultivars. These discrepancies could potentially stem from variations in species and/or cultivar, or may be attributed to differences in the analytical methods employed. Naili, a varietas of *P. salicina* Lindl., originated in the Fujian province of China and has emerged as one of the prominent plum cultivars in that region. Yinhongli and Yuhuangli are locally esteemed varieties in the Sichuan and Ningxia provinces of China, respectively [25]. Therefore, these indigenous cultivars/varieties can serve as excellent breeding parents for producing high carotenoid content.

Concerning the individual carotenoids present in the skin of the plum cultivars tested, lutein was found to be the predominant carotenoid (Figure 4B), which is consistent with previous findings using other different plum cultivars [26]. However, the profiles of the less abundant carotenoids varied considerably (Figure 4C–F). Five carotenoids (lutein, zeaxanthine, β-cryptoxanthin, α-carotene, and β-carotene) were identified as compounds contributing to the carotenoid profiles among all of the plum cultivars tested (Figure 4B–F). It is widely recognized that these five carotenoids are commonly found in fruit and vegetable crops [10,14]. The carotenoids found in the flesh tissue of most of the cultivars were in the forms of lutein, β-carotene, and zeaxanthin, with β-carotene being the predominant compound, except for Dapple Dandy (Figure 4B–F). The presence of these less prevalent carotenoids can be regarded as a distinguishing characteristic among plum cultivars. Consequently, these cultivars are especially useful for exploring the mechanism of individual carotenoid accumulation in plums, because certain carotenoids were absent in certain tested plum cultivars.

The analysis of the carotenoid biosynthetic genes using qRT-PCR revealed a positive correlation between the expressions of *PSY*, *LCYB*, and *LCYE* and the contents of lutein, zeaxanthine, and total carotenoids. Conversely, the expressions of *PDS* and *CRTISO* showed a negative correlation with the contents of lutein, zeaxanthine, and total carotenoids (Figure 5, Table 2). These results here provide evidence that *PSY*, *LCYB*, *LCYE, PDS*, and *CRTISO* are key coordinated regulation genes contributing to total and individual carotenoid accumulation in plums. The balanced expressions of these genes are determinants of carotenoid accumulation in different plum cultivars. It is imperative to investigate the carotenoid profiles and examine the variations in carotenoid biosynthetic gene expressions among different plum cultivars in order to facilitate the development of plum germplasm resources that prioritize cultivars with elevated carotenoid content.

## 4. Materials and Methods

### 4.1. Plant Methods and Sampling

This study was conducted using five-year-old bearing trees in a commercial plum grove in Yanpin Village, Jiuxiang Town, Hanyuan County, Ya’an City, Sichuan Province, China (latitude: 31°28′34″ N and longitude: 103°37′18″ E). The trees were grafted on the same rootstock (*Amygdaluspersica* L. Batsch), had a planting density of 3 m × 3 m, and were subjected to identical horticultural management and pest control operations. The cultivar specifications can be found in Table 1. A total of thirty fruit samples were randomly collected from the upper, middle, and lower canopy of each tree. These fruit samples, which amounted to a total of 90 fruits, were considered as one biological replicate. Each cultivar had three biological replicates. Fruit samples were collected at the stage of physiological maturity, determined by size, color, and firmness, indicating their readiness for consumption.

### 4.2. Chemicals and Reference Standards

High-performance liquid chromatography (HPLC)-grade authentic standard reagents, including α-carotene, β-carotene, lutein, zeaxanthin, and β-cryptoxanthin, were purchased from Extrasynthese (Genay, France). HPLC-grade methanol, acetone, and methyl tert-butyl ether were purchased from Beijing Solarbio Science & Technology Co. Ltd. (Beijing, China). All aqueous solutions were prepared using ultra-high-purity water (18.2 MΩ. cm) obtained from a Milli-Q water purification system (Millipore Corporation, Bedford, MA, USA), filtered through a 0.22 μm filter.

### 4.3. Determination of Fruit Weight, Shape Index, and Color Measurement

Fresh weight (FW) was determined by averaging the weights of 30 fruits in each biological replicate. The fruit shape index was calculated by dividing the vertical length of the fruit by its transverse diameter. Measurements of flesh color were conducted using a CM-2600d spectrophotometer (Konica Minolta, Tokyo, Japan), with three points measured around the equatorial plane of the fruit. The resulting ‘L*’ value represents lightness, while ‘a*’ and ‘b*’ indicate color directions, with ‘+a*’ and ‘−a*’ representing the red and green axes, respectively, and ‘+b*’ and ‘−b*’ representing the yellow and blue axes, respectively.

### 4.4. Extraction and Quantification of Carotenoids Using HPLC

The frozen samples were ground into a fine powder using a pestle and mortar in liquid nitrogen. Subsequently, 1.0 g of the pulverized samples was homogenized in 5.0 mL of a 1% (*v*/*v*) solution of butylated hydroxytoluene (BHT)–acetone. The resulting mixture underwent ultrasonical extraction for a duration of 1 h at 4 °C in darkness, followed by centrifugation at a speed of 10,000 r/min for 10 min. The supernatants were subjected to filtration using a 0.22 μm filter prior to the determination of both total and individual carotenoids.

The quantification of total carotenoids was performed using a MAPADA UV-1200 spectrophotometer (Meipuda Instrument Co. Ltd., Shanghai, China), with an absorbance measurement at a wavelength of 470 nm. The concentration of the total carotenoids was calculated using the following formula:

Total carotenoid content (mg/g) = A × V × N × 10/(m × 2500), where A represents the absorbance value at 470 nm of the sample, V represents the sample volume, N represents the dilution multiple, and m represents the fresh weight (FW, g).

Carotenoid compositions were determined using an Agilent 1260 HPLC system (Agilent Technologies, Pala Alto, CA, USA) equipped with a diode array detector (Agilent) and YMC-C_30_ column (250 mm × 4.6 mm, 5 μm; YMC, Wilmington, NC, USA). An isocratic elution method was employed with a mobile phase consisting of 30:70 (*v*/*v*) methyl tert-butyl ether and methanol, at a flow rate of 1.0 mL min^−1^. The injection volume utilized in this experiment was 20 μL, while the oven temperature was maintained at 25 °C and the detector wavelength was at 450 nm. Each sample was subjected to triplicate measurements.

### 4.5. Quantitative Real-Time Polymerase Chain Reaction (qRT-PCR)

The specific primers targeting the genes associated with carotenoid biosynthesis can be found in Table 3 and were procured from Sangon Biotech (Shanghai) Co., Ltd. (Shanghai, China), The *PsActin* gene served as the internal reference gene. The total RNA was extracted using TRIzol reagent (Invitrogen, Carlsbad, CA, USA), and the subsequent purification of mRNA was carried out using a TURBO DNA-free kit (Ambion, Austin, TX, USA). Complementary DNA synthesis was performed using a PrimeScript™ RT reagent kit with gDNA Eraser (Perfect Real Time) (TaKaRa, Dalian, China). The qRT-PCR analysis was conducted using a CFX96 Touch Real-Time PCR C1000 Thermal Cycler system (Bio-Rad, Hercules, CA, USA) and an SYBR^®^ Premix Ex Tag^™^ II (Tli RNaseH Plus) (Takana, Dalian, China), according to the manufacturer’s instructions. The amplification programs consisted of an initial denaturation step at 95 °C for 30 s, followed by 40 cycles of denaturation at 95 °C for 30 s and annealing/extension at 58 °C for 30 s. Each biological replicate was subjected to triplicate PCR amplification. The relative expression levels of the target genes were normalized to those of the internal reference gene (*PsActin*) using the 2^−(ΔΔCt)^ method.

### 4.6. Statistical Analyses

The results, obtained from three independent biological replicates, were expressed as the mean ± standard deviation (SD). The statistical differences among cultivars were evaluated using one-way analysis of variance (ANOVA) with Duncan’s multiple range tests at a significance level of *p* < 0.05 using SPSS v. 20.0 (SPSS Inc., Chicago, IL, USA). The correlation between carotenoid content and relative gene expression was determined using Person’s correlation coefficient at a significance level of *p* < 0.05.

## 5. Conclusions

This study aimed to analyze the carotenoid profiles of ten plum cultivars, including the identification and quantification of individual carotenoids, as well as total and individual carotenoids. The results showed that total and individual carotenoid contents in the skin tissues were significantly higher compared to those in flesh samples among all of the tested plum cultivars. The carotenoid content exhibited significant variation depending on cultivars, with Naili having the highest total carotenoid content, followed by Yinhongli and Yuhuangli. The expressions of *PSY*, *LCYB*, and *LCYE* demonstrated a positive correlation with lutein, zeaxanthine, and total carotenoid contents, while the expressions of *PDS* and *CRTISO* exhibited a negative correlation with lutein, zeaxanthine, and total carotenoid contents. It is crucial to determine the carotenoid profiles and investigate disparities in carotenoid biosynthetic gene expressions among plum cultivars in order to facilitate plum germplasm resource innovation aimed at developing cultivars with high carotenoid content.

## Figures and Tables

**Table 1:** Plum cultivars used in this study.

No.	Fruit Phenotype	Fruit Transverse Section	Common Name	Scientific Name
1	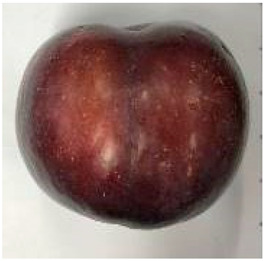	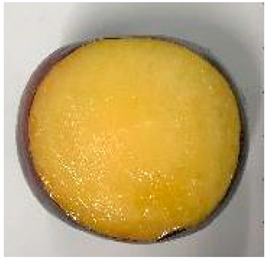	Black Diamond	*Prunus salicina* Lindl. cv. ‘Black Diamond’
2	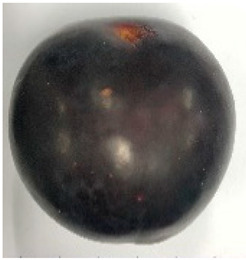	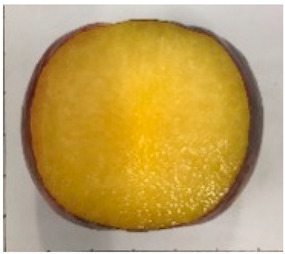	Black Amber	*Prunus salicina* Lindl. cv. ‘Black Amber’
3	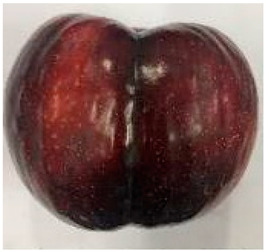	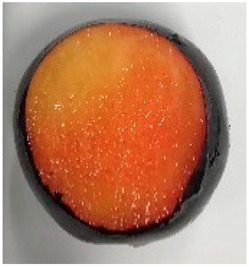	Angeleno	*Prunus salicina* Lindl. cv. ‘Angeleno’
4	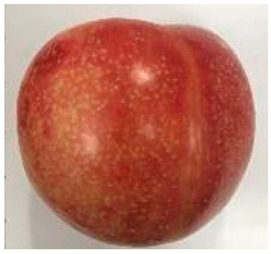	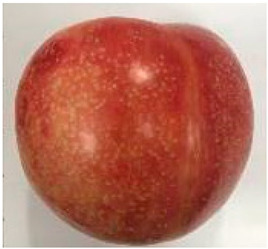	Dapple Dandy	*Prunus salicina* Lindl. cv. ‘Dapple Dandy’
5	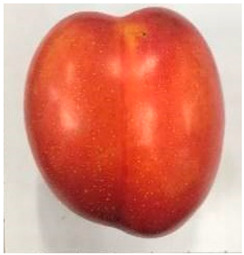	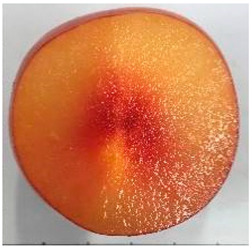	Friar	Prunus salicina Lindl. cv. ‘Friar’
6	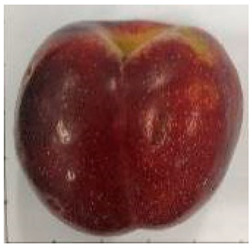	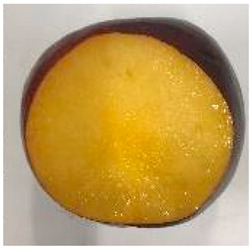	Ooishi-wase	Prunus salicina Lindl. cv. ‘Ooishi-wase’
7	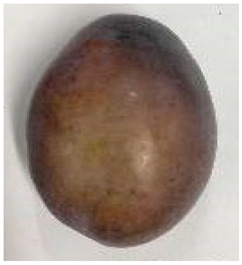	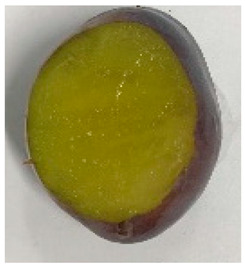	Empress	*Prunus domestica* L cv. ‘Empress’
8	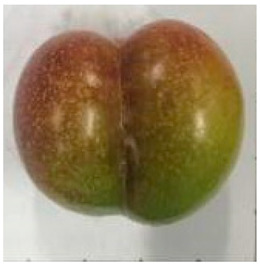	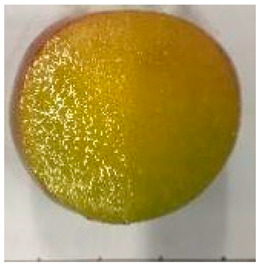	Yinhongli	*Prunus salicina* Lindl. cv. ‘Yinhongli’
9	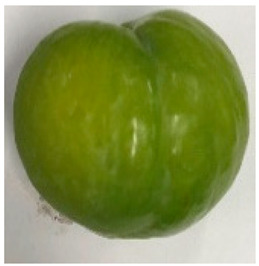	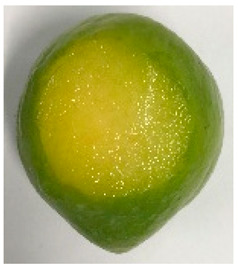	Naili	*Prunus salicina* Lindl. var. cordata; Y. He and J. Y. Zhang
10	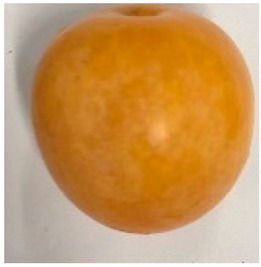	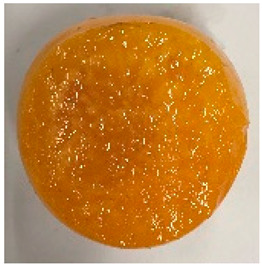	Yuhuangli	*Prunus salicina* Lindl. cv. ‘Yuhuangli’

**Table 2:** Correlation analysis of carotenoid content and gene expression in peels of different plum cultivars.

Gene Name	Lutein	Zeaxanthine	β-Cryptoxanthin	α-Carotene	β-Carotene	Total Carotenoid
*DXS*	−0.301	−0.123	−0.043	−0.354	−0.280	−0.365
*DXR*	0.261	0.396	0.044	0.061	−0.136	0.132
*PSY*	0.574 **	0.614 **	0.093	0.213	0.052	0.444 *
*PDS*	−0.484 *	−0.464 *	−0.075	0.375	0.235	−0.168
*ZDS*	0.131	0.312	0.207	0.641**	0.341	0.358
*ZISO*	0.311	0.368	0.018	0.392	0.110	0.316
*CRTISO*	−0.566 **	−0.700 **	−0.031	0.179	0.224	−0.264
*LCYB*	0.422 *	0.572 **	0.172	0.447*	0.149	0.432 *
*LCYE*	0.801 **	0.699 **	0.119	0.161	0.236	0.666 *
*ECH*	0.056	−0.200	−0.232	−0.201	−0.420 *	−0.224
*BCH*	0.175	−0.010	0.035	0.480 *	0.500 *	0.400
*VDE*	0.151	0.177	0.051	0.616 **	0.340	0.345
*ZEP*	0.354	0.589 **	−0.157	0.294	0.029	0.301
*NSY*	0.106	0.306	−0.072	0.097	−0.202	0.001
*CCD4*	0.149	0.028	−0.521 **	−0.489 *	−0.437 *	−0.207
*NCED*	−0.230	0.033	−0.570 **	−0.354	−0.429 *	−0.407 *

Note: * and ** represent the *p* values less than 0.05 and 0.01, respectively.

**Table 3:** Primers used for quantitative real-time polymerase chain reaction in this study.

Gene Name	Forward Primer Sequence	Reverse Primer Sequence
	(5′-3′)	(5′-3′)
*Actin*	GCAGACAGGATGAGCAAGGAGATTAC	TCTGTTGGAAGGTACTGAGGGATG
*DXS*	TGCCTCAACACTCCAAA	CCGTCTGCGTTACCAG
*DXR*	GAGAACCCAGATAAATT	CCTGATCCACAAGAAG
*PSY*	TGGAAAGGTCACAGACAAGT	TGCTGGCTTCACTCAACTC
*PDS*	TGAAGTTTTACCAGCACCCT	AACATAAGCCTGCCCACCA
*ZDS*	GGTTCACTCCTCCAATG	CTTCGTCACTCTTGCTAT
*ZISO*	TACCGCTGGCTGTTAGT	TGAAAGTGGACGGATAGA
*CRTISO*	AGCATTCCAACTGTTCTTGA	TTCTTTGCCTCATAGTCCTT
*LCYB*	AATCCAGGTTACCAAGTG	GTTCAAGTGTGAGTCTCT
*LCYE*	ATCCAGCCACAGGGTA	CCAGAGGGTGTTCCAT
*ECH*	GGAGTGAAGCGGTTGT	GAGGACTTGGGCTCTATT
*BCH*	GTGAGGCGTGAGGTGCT	CCTTCCCATCATACTCTGTG
*VDE*	GGGGAATTTGTCTTGG	GGCTGATTTGGGTCTT
*ZEP*	ACCGAGTATTTTTGGGGCAC	GCAGCCTTTCCTTTTTACCG
*NSY*	TTCTGGCACCTAAAGC	AATCAACCGTAGCGTCT
*CCD4*	GCTAATGGCTTTGGTCAG	TGGCGTCCCAGAGTTT
*NCED*	GCGAGCCTCTGTTTCTGC	CTCCGATTTCCACTCCTTCT

## Data Availability

All data supporting the findings of this study are available within the paper.

## References

[1] Potter D., Eriksson T., Evans R.C., Oh S., Smedmark J.E.E., Morgan D.R., Kerr M., Robertson K.R., Arsenault M., Dickinson T.A. (2007). Phylogeny and classification of Rosaceae. Plant Syst. Evol..

[2] Sottile F., Caltagirone C., Giacalone G., Peano C., Barone E. (2022). Unlocking plum genetic potential: Where are we at?. Horticulturae.

[3] Zhebentyayeva T., Shankar V., Scorza R., Callahan A., Ravelonandro M., Castro S., DeJong T., Saski C.A., Dardick C. (2019). Genetic characterization of worldwide *Prunus domestica* (plum) germplasm using sequence-based genotyping. Hortic. Res..

[4] Drogoudi P., Pantelidis G. (2022). Phenotypic variation and peel contribution to fruit antioxidant contents in european and japanese plums. Plants.

[5] Liu W., Liu D., Zhang A., Feng C., Yang J., Yoon J., Li S. (2007). Genetic diversity and phylogenetic relationships among plum germplasm resources in China assessed with inter-simple sequence repeat markers. J. Am. Soc. Hortic. Sci..

[6] Roussos P.A., Efstathios N., Intidhar B., Denaxa N.K., Tsafouros A., Simmonds M., Preedy V. (2016). Plum (*Prunus domestica* L. and *P. salicina* Lindl.). Nutritional Composition of Fruit Cultivars.

[7] Zhang H., Pu J., Tang Y., Wang M., Tian K., Wang Y., Luo X., Deng Q. (2022). Changes in phenolic compounds and antioxidant activity during development of ‘Qiangcuili’ and ‘Cuihongli’ Fruit. Foods.

[8] Deng H., He R., Xia H., Xu N., Deng Q., Liang D., Lin L., Liao L., Xiong B., Xie X. (2022). Ultra-HPLC-MS pseudo-targeted metabolomic profiling reveals metabolites and associated metabolic pathway alterations in Asian plum (*Prunus salicina*) fruits in response to gummosis disease. Funct. Plant Biol..

[9] Nisar N., Li L., Lu S., Khin N.C., Pogson B.J. (2015). Carotenoid metabolism in plants. Mol. Plant.

[10] Sun T., Rao S., Zhou X., Li L. (2022). Plant carotenoids: Recent advances and future perspectives. Mol. Hortic..

[11] Eggersdorfer M., Wyss A. (2018). Carotenoids in human nutrition and health. Arch. Biochem. Biophys..

[12] Crupi P., Faienza M.F., Naeem M.Y., Corbo F., Clodoveo M.L., Muraglia M. (2023). Overview of the potential beneficial effects of carotenoids on consumer health and well-being. Antioxidants.

[13] Meléndez-Martínez A.J., Mapelli-Brahm P., Stinco C.M. (2018). The colourless carotenoids phytoene and phytofluene: From dietary sources to their usefulness for the functional foods and nutricosmetics industries. J. Food Compos. Anal..

[14] Sun T., Yuan H., Cao H., Yazdani M., Tadmor Y., Li L. (2018). Carotenoid metabolism in plants: The role of plastids. Mol. Plant.

[15] Rodríguez-Villalón A., Gas E., Rodríguez-Concepción M. (2009). Phytoene synthase activity controls the biosynthesis of carotenoids and the supply of their metabolic precursors in dark-grown *Arabidopsis* seedlings. Plant J..

[16] Breitenbach J., Sandmann G. (2005). ζ-Carotene cis isomers as products and substrates in the plant poly-cis carotenoid biosynthetic pathway to lycopene. Planta.

[17] Ye Z.W., Jiang J.G. (2010). Analysis of an essential carotenogenic enzyme: ζ-carotene desaturase from unicellular alga dunaliella salina. J. Agric. Food Chem..

[18] Isaacson T., Ronen G., Zamir D., Hirschberg J. (2002). Cloning of tangerine from tomato reveals a carotenoid isomerase essential for the production of β-carotene and xanthophylls in plants. Plant Cell.

[19] Liao L., Li Y., Lan X., Yang Y., Wei W., Ai J., Feng X., Chen H., Tang Y., Xi L. (2023). Integrative analysis of fruit quality and anthocyanin accumulation of plum cv. ‘Cuihongli’ (*Prunus salicina* Lindl.) and its bud mutation. Plants.

[20] Sahamishirazi S., Moehring J., Claupein W., Graeff-Hoenninger S. (2017). Quality assessment of 178 cultivars of plum regarding phenolic, anthocyanin and sugar content. Food Chem..

[21] Saini R.K., Nile S.H., Park S.W. (2015). Carotenoids from fruits and vegetables: Chemistry, analysis, occurrence, bioavailability and biological activities. Food Res. Int..

[22] Yuan H., Zhang J., Nageswaran D., Li L. (2015). Carotenoid metabolism and regulation in horticultural crops. Hortic. Res..

[23] Díaz-Mula H.M., Zapata P.J., Guillén F., Martínez-Romero D., Castillo S., Serrano M., Valero D. (2009). Changes in hydrophilic and lipophilic antioxidant activity and related bioactive compounds during postharvest storage of yellow and purple plum cultivars. Postharvest Biol. Technol..

[24] Vizzotto M., Cisneros-Zevallos L., Byrne D.H., Ramming D.W., Okie W.R. (2007). Large variation found in the phytochemical and antioxidant activity of peach and plum germplasm. J. Am. Soc. Hortic. Sci..

[25] Yao L., Liang D., Xia H., Pang Y., Xiao Q., Huang Y., Zhang W., Pu C., Wang J., Lv X. (2023). Biostimulants promote the accumulation of carbohydrates and biosynthesis of anthocyanins in ‘Yinhongli’ plum. Front. Plant Sci..

[26] Kaulmann A., Jonville M.C., Schneider Y.J., Hoffmann L., Bohn T. (2014). Carotenoids, polyphenols and micronutrient profiles of Brassica oleraceae and plum varieties and their contribution to measures of total antioxidant capacity. Food Chem..

